# Evaluation of Anti-Biofilm Property of Zirconium Oxide Nanoparticles on Streptococcus mutans and Enterococcus faecalis: An In Vitro Study

**DOI:** 10.7759/cureus.77199

**Published:** 2025-01-09

**Authors:** Anu Priya Guruswamy Pandian, Anil Kumar Ramachandran, Priyanka Kodaganallur Pitchumani, Blessy Mathai, Davis C Thomas

**Affiliations:** 1 Endodontics, Private Practice, Los Angeles, USA; 2 Endodontics, Ragas Dental College and Hospital, Chennai, IND; 3 Periodontics, University of Iowa College of Dentistry, Iowa, USA; 4 General Dentistry, Private Practice, Newark, USA; 5 Orofacial Pain, Eastman Institute for Oral Health, Rochester, USA; 6 Diagnostic Sciences, Rutgers School of Dental Medicine, Newark, USA

**Keywords:** anti-biofilm, endodontic treatment, intracanal irrigants, secondary endodontic infections, zirconium oxide nanoparticles

## Abstract

Introduction

The prevalence of dental caries and endodontic infections caused by bacteria such as *Streptococcus mutans* and *Enterococcus faecalis* poses a significant challenge in root canal treatment. These pathogens exhibit significant resistance to antimicrobial treatments due to their ability to form biofilms, complex microbial communities encased in a self-produced extracellular polymeric substance (EPS). Despite advancements in treatment strategies, the failure rate remains high due to the difficulty in completely eradicating microbial populations within the complex root canal system. The increasing prevalence of antibiotic resistance has driven the need for novel antimicrobial agents and strategies to target biofilms. In this study, we evaluated the potential of zirconium oxide nanoparticles (ZrO_2 _NPs) in eradicating biofilms formed by *S. mutans* and *E. faecalis*, two key pathogens involved in these infections.

Materials and methods

ZrO_2 _NPs were characterized and evaluated for their antimicrobial properties against *E. faecalis *and *S. mutans *biofilms. The nanoparticles were characterized using scanning electron microscopy and energy-dispersive X-ray spectroscopy. Biofilm quantification was performed using a crystal violet staining assay. The minimum biofilm eradication concentrations of ZrO_2 _NPs against the bacterial strains were determined.

Results

ZrO_2 _NPs used in our study were found to have zirconium predominantly with no impurities. Also, the average particle size was less than 30 nm with a spherical shape arranged in clusters. These characterizations were performed considering the vital role of these properties in obtaining the desired antibacterial/anti-biofilm properties of nanoparticles. ZrO_2 _NPs were capable of eradicating the biofilm formed by both *S. mutans* and *E. faecalis* at concentrations greater than 22.8 μg/mL. The results suggest that ZrO_2 _NPs could be used as a novel approach for combating biofilm-related infections.

Conclusion

Our findings suggest that ZrO_2 _NPs have the potential to be a promising antibiofilm agent in root canal treatment, offering a new approach to combat these persistent infections. Further research is warranted to explore the full potential of nanomaterials in improving treatment outcomes in endodontic infections.

## Introduction

Dental caries is a common oral disease caused by biofilms, resulting in inflammation of the pulp and surrounding tissues [1]. Studies have shown that *Streptococcus mutans* (*S. mutans*) is predominantly responsible for caries initiation due to its aciduric and acidogenic properties. In addition, glucan synthesized by *S. mutans* to form biofilms can potentially act as a protective barrier against antibacterial agents [2,3]. Caries progression leading to pulpal infections can provide a selective habitat for bacteria and their by-products to cause endodontic infections [4,5]. Primary endodontic infections are polymicrobial, whereas secondary endodontic infections predominantly comprise bacterial species that possess antimicrobial resistance and survive ecological changes [6]. Studies have shown that *Enterococcus faecalis* (*E. faecalis*) is the most commonly isolated species from secondary endodontic infections due to its ability to survive nutrient-depleted environments [7].

Endodontic treatment aims to eliminate or reduce the microbial population within the root canal system [8]. However, complete eradication of microbes is difficult, owing to the complex anatomy of the root canal system and growing antimicrobial resistance [9,10]. Current cleaning and shaping techniques are unable to eliminate bacterial biofilms within the anatomic complexities and uninstrumented portions of the root canal system, which presents another significant challenge in root canal treatment [11,12].

The current level of evidence shows that failure rates in endodontic treatments range from approximately 15% to 25% despite the advancements [13]. The shortcomings of the current antibiofilm strategies in root canal treatment has directed research on the applicability nanomaterials in this field [14].

Among the most studied metal oxide nanoparticles, we chose zirconium oxide nanoparticles (ZrO_2_ NPs) because of their biocompatibility and extensive use in the field of dentistry. There are no published studies evaluating the ability of ZrO_2_ NPs to eradicate biofilm formed by *S. mutans*, the primary organism responsible for the initiation of caries, and *E. faecalis*, a highly resistant endodontic pathogen. The purpose of this study was to evaluate the minimum biofilm eradication concentration (MBEC) of ZrO_2_ NPs against *S. mutans* and *E. faecalis*.

## Materials and methods

ZrO_2_ NPs were obtained from Nano Research Lab (Jamshedpur, Jharkhand, India). Standard ATCC strains of *E. faecalis *(ATCC 29212) and *S. mutans* (ATCC 25175) were procured from Sigma Aldrich (Chemicals Private Ltd., Bengaluru, Karnataka, India) and sub-cultured onto brain heart infusion agar (BHIA) plate (Ref. MV 210-500G, HiMedia, Thane, Maharashtra, India) at 37oC for 24 hours. All manufacturer guidelines and instructions regarding the handling and care of the materials were strictly adhered to.

Characterization of zirconium oxide nanoparticles

The morphology and chemical composition of powdered ZrO_2_ NPs were analyzed using scanning electron microscopy (SEM) and energy-dispersive X-ray spectroscopy (EDAX), respectively.

Biofilm quantification of *E. faecalis *and *S. mutans* by crystal violet staining assay

The growth conditions for biofilm formation and crystal violet staining were adapted according to the modified method described by Melo et al. [15]. The strains were initially grown on BHIA at 37°C for 24 hours. Two to three identical colonies of standard strain were further sub-cultured in RPMI 1640 broth medium without bicarbonate, buffered to pH 7.0 with 3-(N-morpholino) propane sulfonic acid (MOPS) overnight. The cell cultures were harvested, washed twice with phosphate-buffered saline (PBS), and adjusted to ~108 CFU/mL in RPMI 1640. A total of 100 µL of this cell suspension was seeded into the respective wells of polystyrene, round-bottom, 96-well microtiter plates, and the plates were incubated at 37°C for 2 hours at 75 rpm for the planktonic cells to adhere onto the inner walls of the microtiter plate wells. Following the attachment phase, non-adherent cells were removed by washing the wells with 150 µL of PBS, and 100 µL of fresh RPMI 1640 medium was added to each well. The plate was incubated at 37°C for 48 hours on a shaker at 75 rpm for the growth and maturation of biofilms. The test medium RPMI 1640 without inoculum was added to the final well of each plate as the negative control.

The biofilms formed after incubation were quantified using the crystal violet staining method. The biofilms were washed twice with 200 µL of PBS, and the plate was dried for 20 minutes at 37°C; subsequently, the biofilms were stained with 110 µL of 0.4% aqueous crystal violet for 45 minutes and then washed thrice with 200 µL of sterile Milli-Q water and left for destaining with 200 µL of 95% ethanol for 45 minutes. A final volume of 100 µL of the destaining solution from each sample was transferred to a new flat bottom plate and measured with a spectrophotometric plate reader at 595 nm. The absorbance values of the negative control were subtracted from the values of the test wells to minimize background interference. The biofilm production quantities were reported as the arithmetic mean of the highest absorbance values of the test wells. The biofilms formed were classified into strong, moderate, and weak based on the spectrophotometric absorbance at 595 nm relative to the corresponding absorbance of the cell-free medium control.

Crystal violet binds to both cells and the extracellular polymeric substances (EPS) of biofilms, providing a measure of the total biomass. After staining, the dye is solubilized, and absorbance is measured spectrophotometrically, offering a quantitative evaluation of biofilm formation.

Minimum biofilm eradication concentrations of zirconium oxide nanoparticles against *E. faecalis *and *S. mutans*

After 24 hours of biofilm growth, they were washed twice with PBS, and the plates were inverted onto absorbent paper to remove residual buffer prior to exposure with ZrO_2_ NPs. The final dilutions of ZrO_2_ NPs were added to respective wells of the microtiter plate and then incubated for 48 hours at 37°C. Wells containing biofilms, but no drug, served as positive controls for each strain tested. Following the addition of ZrO_2_ NPs, biofilms were washed three times with sterile PBS, and metabolic activity was determined using the XTT reduction assay. A total of 100 μL of XTT was added to each well and incubated at 37°C in the dark for 1 hour. Before reading, 100 μL of reaction mixture was transferred to a clean flat-bottomed microtiter plate and read in a spectrophotometer at 490 nm. The absorbance values of the negative control wells (containing no cells) were subtracted from the values of the test wells to account for any background absorbance.

## Results

Characterization of zirconium oxide nanoparticles

**Figure 1:**
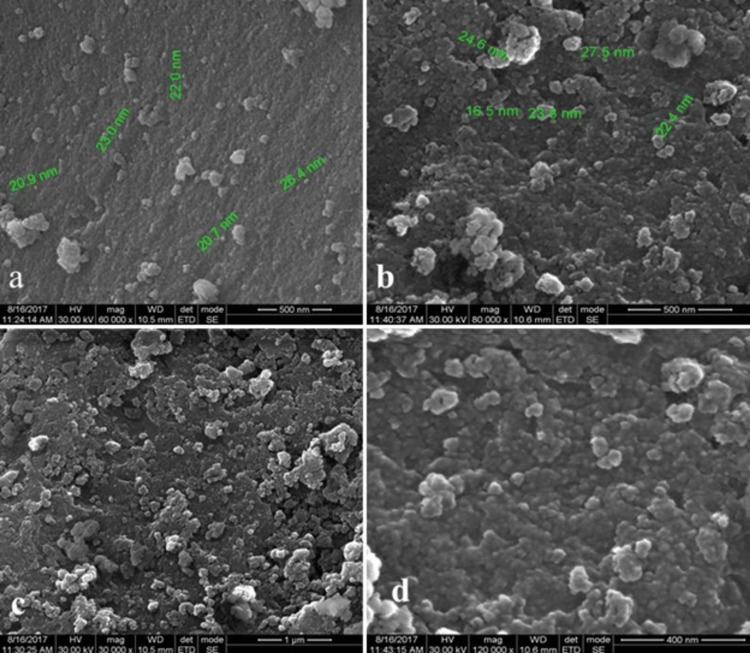
SEM micrographs of ZrO2 NPs The average particle size of ZrO_2_ NPs used in our study is shown in Figures 1a, 1b. Similarly, the shape and arrangement of ZrO_2_ NPs can be interpreted from Figures 1c, 1d. SEM, scanning electron microscopy; ZrO_2_ NPs, zirconium oxide nanoparticles

SEM analysis demonstrated the average particle size of ZrO2 NPs to range from 16.5 nm to 27.5 nm (Figures 1a, 1b) with spherical particles arranged in clusters (Figures 1c, 1d).

**Figure 2:**
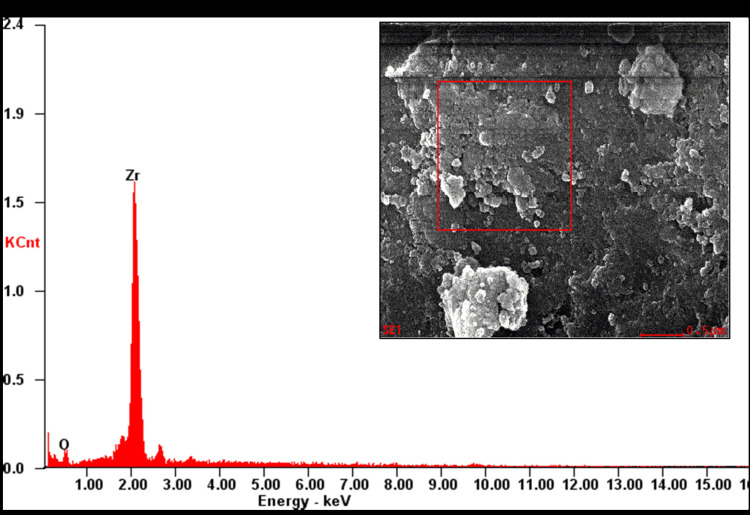
Elemental analysis of powdered ZrO2 NPs using EDAX The peak in the graph shows that zirconium was the predominant component in the powdered ZrO2 NPs used in our study with no impurities or additives. EDAX, energy-dispersive X-ray spectroscopy; ZrO2 NPs, zirconium oxide nanoparticles

Elemental analysis of ZrO_2_ NPs was done using EDAX, and the peak values showed that the predominant element in the sample was zirconium (Figure 2).

Determination of growth conditions and biofilm quantification of *E. faecalis* and *S. mutans* by crystal violet staining assay

**Figure 3:**
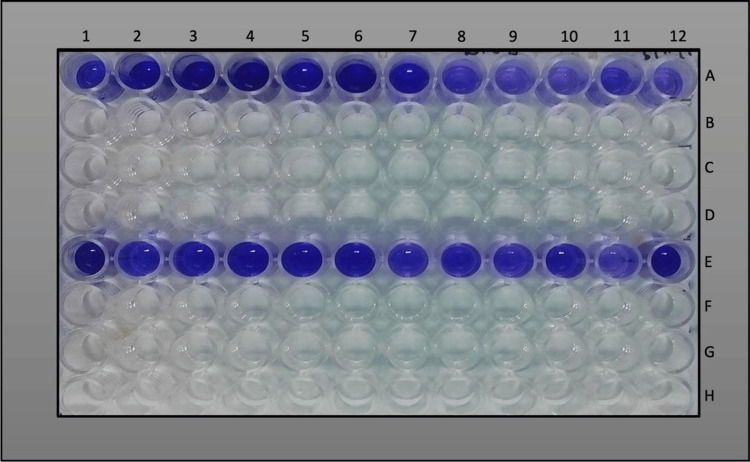
Crystal violet assay for S. mutans and E. faecalis Numbers 1-12 represent the wells in the petri dish. Well 12: test medium RPMI 1640 (cell culture medium) without inoculum acts as a negative control. Well A and E: contain S. mutans and E. faecalis inoculum, respectively.

The ability of *S. mutans* and *E. faecalis* to form biofilm was determined using crystal violet staining assay (Figure 3).

The spectrophotometric values of the biofilms formed by *S. mutans* and *E. faecalis* are listed in Table 1.

**Table 1:** Spectrometric values for crystal violet assay read at 595nm Dense growth where OD was not read at 590 nm. Wells 1-12 indicate the wells in the Petri dish. A and E indicate the wells in which* S. mutans *and *E. faecalis *inoculum were cultured and stained. ***Dense biofilm formation that could not be read spectrophotometrically. OD, optical density

Spectrometric readings (optical density)													
		1	2	3	4	5	6	7	8	9	10	11	12
S. mutans	A	***	1.931	3.086	***	***	***	1.522	2.046	1.342	2.085	0.816	***
E. faecalis	E	2.19	***	***	***	***	***	***	1.17	0.792	0.725	1.433	0.835

The density of biofilm formed by *S. mutans* and *E. faecalis* was calculated by taking the arithmetic mean of the three highest OD_595nm_ values and comparing it with the inoculum OD_595nm_. The arithmetic mean of optical density values for both *S. mutans *and *E. faecalis* is presented in Table 2. The mean for *S. mutans* was 2.021 with a standard deviation of 0.08 and that for *E. faecalis* was 1.590 with a standard deviation of 0.53.

**Table 2:** Calculation of biofilm formation The arithmetic mean of the highest OD values from spectrometric readings in *S. mutans *and *E. faecalis *is calculated OD, optical density

Bacterial strain	Average of OD_595nm_		Evaluation with inoculum OD_595nm_
S. mutans	2.046 + 2.085 + 1.931	2.021	>0.8
	3		
E. faecalis	2.190 + 1.170 + 1.433	1.590	>0.8
	3		

Based on the spectrophotometric absorbance at 595 nm in relation to the corresponding absorbance of the cell-free control medium, biofilm formation was classified into four types, as shown in Table 3: <0.2, non-adherent (-); 0.2 ≤ 0.4, weakly adherent (₊); 0.4 ≤ 0.8, moderately adherent (₊ ₊); and >0.8, strongly adherent (₊ ₊ ₊).

**Table 3:** Biofilm quantification using the crystal violet method Note: inoculum OD_595nm_ was approximately 0.2

Strain	OD_595nm_	Interpretation
S. mutans: average of OD_595nm_	2.021	Strong (+++)
E. faecalis: average of OD_595nm_	1.590	Strong (+++)

Minimum biofilm eradication concentrations of zirconium oxide nanoparticles against E. faecalis and S. mutans

**Figure 4:**
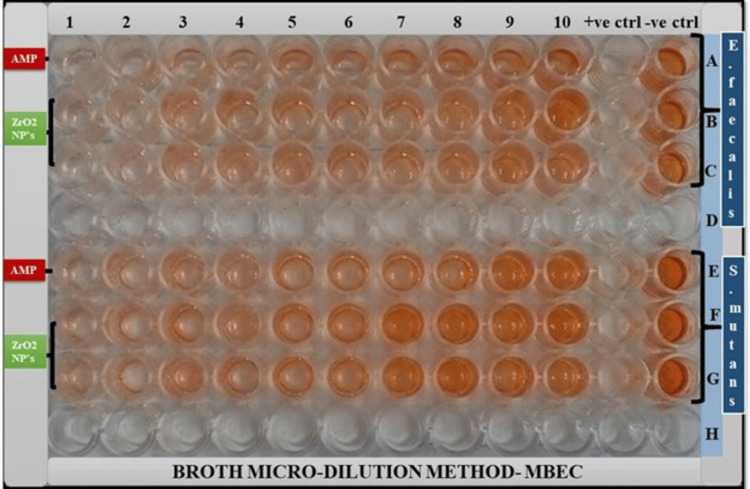
Serial dilutions of ZrO2 NPs against S.mutans and E. faecalis biofilm Wells 1-10 indicate wells in a microtiter plate. Wells A-D contains *E. faecalis *biofilm, in which A is treated with ampicillin, B and C are treated with ZrO_2_ NPs, and D is untreated. Wells E-H contains *S. mutans *biofilm, in which E is treated with ampicillin, F and G are treated with ZrO_2_ NPs, and H is untreated. Positive control: contains biofilms, but no drug. Negative control: contains no bacterial strain.

MBEC for ZrO_2_ NPs is defined as the lowest drug concentration which inhibits the metabolic activity of *S. mutans *and *E. faecalis *by 50% (MBEC_50_) and 80% (MBEC_80_), respectively, compared to drug-free growth control well. Two replicate tests were conducted with each of the isolates (Figure 4).

The visual turbidity of the wells for MBEC is spectrometrically read at 490 nm (Table 4).

**Table 4:** Spectrometric values for MBEC read at 490 nm MBEC, minimum biofilm eradication concentration

Spectrometric readings (optical density)														
			1	2	3	4	5	6	7	8	9	10	11	12
E. faecalis	Ampicillin	A	0.161	0.273	0.200	0.241	0.216	0.267	0.288	0.189	0.442	0.498	0.147	0.425
		B	0.093	0.149	0.179	0.231	0.267	0.209	0.235	0.202	0.182	0.259	0.068	0.331
	Zr0_2_ NPs	C	0.135	0.121	0.146	0.131	0.182	0.218	0.229	0.188	0.101	0.152	0.064	0.222
		D	-	-	-	-	-	-	-	-	-	-	-	-
S. mutans	Ampicillin	E	0.249	0.491	0.258	0.172	0.501	0.476	0.508	0.490	0.259	0.253	0.047	0.449
		F	0.174	0.836	0.603	0.267	0.427	0.434	0.111	0.552	0.332	0.367	0.041	0.359
	Zr0_2_ NPs	G	0.162	0.632	0.258	0.120	0.337	0.416	0.206	0.364	0.384	0.210	0.058	0.471
		H	-	-	-	-	-	-	-	-	-	-	-	-

MBEC was then calculated using the following formula:

$MBEC = \frac{OD_{(untreated)}-OD_{(test)}}{OD_{(untreated)}-OD_{(blank)}}\times 100$

where OD_(untreated)_ is OD in positive media control wells with only media and the bacterial strain, OD_(test)_ is OD in wells treated with ZrO_2_ NPs against *E. faecalis *and *S. mutans*, and OD_(blank)_ is OD in negative growth control wells with only culture media.

The optical density values were substituted in the formula and the percentage thus obtained was taken close to 50% and 80% to calculate MIC_50_ and MIC_80_, respectively. The concentration at which ZrO_2_ NPs eradicated 50% and 80% of *S. mutans* and *E. faecalis *biofilm, respectively, is listed in Table 5.

**Table 5:** Calculation of MBEC50 and MBEC80 The optical density values were substituted in the formula mentioned in the table. The percentage obtained was taken close to 50% and 80% to calculate MBEC_50_ and MBEC_80_, respectively. MBEC_50_: minimum concentration at which approximately 50% of the biofilm is eradicated. MBEC_80_: minimum concentration at which approximately 80% of the biofilm is eradicated. MBEC, minimum biofilm eradication concentration

Bacterial strain	MBEC_50_		MBEC_80_	
*S. mutans*	0.471 - 0.258	x 100 = 51.3%	0.471 - <0.162	x 100 = >74.4%
	0.471 - 0.056		0.471 - 0.056	
*E. faecalis*	0.331 - 0.146	x 100 = 55.1%	0.331 - 0.135	x 100 = 75%
	0.331 - 0.068		0.331 - 0.068	

## Discussion

The structural, metabolic, and chemical interactions between microorganisms in the oral biofilm play an important role in maintaining community homeostasis and provide a balanced equilibrium with host defenses for maintaining the integrity of oral tissues. Disease occurs when this balance is disturbed to the benefit of the biofilm [16]. *Streptococcus mutans* behave as an opportunistic bacteria by proliferating under favorable conditions and initiating the formation of dental caries [17]. The acid production of the bacterium serves as a virulence factor because the acidic products formed during the metabolism of dietary carbohydrates are essential for the development of caries. Synthesis of insoluble extracellular polysaccharides from sucrose through glucosyltransferases is also considered another important virulence factor because it not only facilitates the adhesion and accumulation of the organism on the tooth surface but also provides protection against host immune defenses and increased resistance against antibiotics and gene expression [18,19]. This combination of virulence properties allows *S. mutans* to colonize the surface of the tooth and modify the non-pathogenic communities to highly cariogenic dental biofilm, which ultimately leads to caries formation [20]. Various treatment strategies have been developed to minimize or interrupt the colonization of *S. mutans*, thereby preventing dental caries.

Microbes in secondary infection resist intracanal disinfection procedures and adapt to changing environments to cause persistent intra-radicular infection. *Enterococcus faecalis* is the most consistently reported organism in secondary endodontic infection [6,21]. *Enterococcus faecalis *possesses several virulence factors that permit adherence to host cells and extracellular matrix, colonizing the root canal system. The primary cause for *E. faecalis *to be associated with endodontic failure is its ability to invade dentinal tubules and strongly adhere to collagen, which is abundantly present in root dentin and cementum through collagen binding [22].

One of the bases for the failure of endodontic procedures is the difficulty of accessing and cleansing segments of the root canal anatomy [23]. Conventional irrigants, irrigation techniques, and intra-canal medicaments have minimal penetrability into the dentinal tubules, leaving the bacteria within the tubules undisturbed leading to the persistence of infection within the canal space [24]. Therefore, complete disinfection can be achieved by developing newer materials and techniques that improve not only infection control in the main canal lumen but also the entire root canal system [8].

*Streptococcus mutans* and* E. faecalis*, which were considered the predominant species in the development of dental caries and secondary endodontic infections, were selected as the test organisms. Their ability to form biofilms was also evaluated in our study using crystal violet assay as both *S. mutans* and *E. faecalis* possess biofilm mode of bacterial growth as an adaptive process to survive. These sessile biofilm bacteria also show higher antimicrobial resistance compared to their free-floating “planktonic” counterparts, and this resistance has been attributed to the protective barrier provided by the extracellular polymeric matrix (EPM) [6,7]. Our study shows that both *S. mutans *and *E. faecalis* form strongly adherent biofilms. This may emphasize the need for the development of newer preventive and therapeutic agents that are capable of disrupting the EPM.

Minimum biofilm eradication concentrations of zirconium oxide nanoparticles against *E. faecalis* and *S. mutans*

The biofilms formed by *S. mutans *and *E. faecalis* were more resistant to antimicrobial agents than their planktonic forms. ZrO_2 _NPs were capable of disrupting 50% and 80% of biofilms formed by *S. mutans* and *E. faecalis*, respectively. The MBEC80 values for ZrO_2_ NPs against *S. mutans* and *E. faecalis* were observed to be 22.8 μg/mL for each organism. Our previous study demonstrated the antibacterial efficacy of ZrO_2_ NPs against *S. mutans *and *E. faecalis* in their planktonic form [25]. However, higher concentrations were necessary to eradicate the biofilm formed by them compared to their planktonic form. This can be attributed to the negatively charged biofilm EPM, which provides resistance to the penetration of ZrO_2_ NPs. The antibacterial activity of ZrO_2_ NPs can be attributed to the interaction of surface-ionized ions with negatively charged cell membranes. The resulting change in the permeability of the bacterial cell membrane causes subsequent leakage of the cytosol, leading to the death of the bacteria [26,27].

Biofilm acts as a chemical barrier by adsorbing the harmful reactive oxygen species from ZrO_2_ NPs from reaching the cell surface, thereby decreasing the effect of ROS [28]. The disruption of biofilm structure formed by both *S. mutans* and *E. faecalis *after treatment with the ZrO_2_ NPs was evident from our study. As there are only limited studies on the antimicrobial activity of ZrO_2_ NPs, our study proved the anti-biofilm property of ZrO_2_ NPs against *S. mutans* and *E. faecalis*.

Since ZrO_2_ NPs were capable of disrupting the biofilm formed by *S. mutans*, it can be effectively used in formulations for topical antimicrobial oral use, such as dentifrices. Morphological analysis of ZrO_2_ NPs used in our study was found to be smaller than the size of bacterial/eukaryotic cells. This reflects that the reduced size of the nanoparticles can have greater penetration into the biofilm matrix even with a shorter duration of exposure. In addition, it can also deliver active agents into the biofilm, leading to disruption of the bacterial cell wall of *S. mutans* and thereby inhibiting their enzymatic activity and bacterial aggregation [29]. In endodontic infections, bacterial biofilm within the depths of dentinal tubules would remain inaccessible for conventional irrigants, medicaments, and sealers, contributing to the persistence of infection within the root canal system [24]. Therefore, nanoparticles with unique properties such as enhanced surface area, and chemical and biological activity can be used as an adjunct to provide complete disinfection of the root canal system [30].

Study limitations

The ability of ZrO_2_ NPs to eradicate the biofilms formed by *S. mutans* and *E. faecalis* was performed in an in vitro experiment. The oral environment can substantially vary from the ideal conditions of an in vitro study, such as the influence of saliva, multiple species associated with biofilm formation, and the microbial resistance offered by them. This study was an attempt to direct further studies on the anti-biofilm properties of ZrO_2_ NPs for their potential use in future.

Further in vivo studies are recommended to understand the interaction of ZrO_2_ NPs with oral cavity, their toxicity, and other considerable effects of ZrO_2_ NPs.

## Conclusions

ZrO_2_ NPs having an anti-bacterial and anti-biofilm activity against *E. faecalis *can be incorporated into irrigant solutions or intracanal medicaments in an attempt to reach the uninstrumented areas at an effective concentration and volume. Because of higher diffusibility, ZrO_2_ NPs can also diffuse into the biofilm structure and anatomical complexities within the root canals, where the bacteria are harbored. Furthermore, the activities of oral microflora being responsible for oral diseases can be kept to a level consistent with oral health by antimicrobial agent inclusion in dentifrices. However, the potential application of ZrO_2_ NPs in these areas requires further genotoxic studies.
